# Commentary: China's Zero-COVID Approach Depends on Shanghai's Outbreak Control

**DOI:** 10.3389/fpubh.2022.912992

**Published:** 2022-06-14

**Authors:** Ali Cheshmehzangi, Tong Zou, Zhaohui Su

**Affiliations:** ^1^Network for Education and Research on Peace and Sustainability (NERPS), Hiroshima University, Hiroshima, Japan; ^2^Department of Architecture and Built Environment, University of Nottingham Ningbo China, Ningbo, China; ^3^Center on Smart and Connected Health Technologies, Mays Cancer Center, School of Nursing, UT Health San Antonio, San Antonio, TX, United States; ^4^School of Public Health, Southeast University, Nanjing, China

**Keywords:** zero-COVID, Shanghai, COVID-19, pandemic, outbreak control, pandemic impact, pandemic control, global impact

More than any other time before, China's zero-COVID approach depends on the success or failure of one city's outbreak control. The ongoing resurgent outbreak in China's main megacity, Shanghai, is marked as a game-changing situation. The current situation in Shanghai started around mid-March 2022, is even more severe than what happened in Wuhan in the early days of the pandemic. The severity of the situation is not based on the number of death cases or the sudden early shocks of the disease spread. It is more about the political pressure and growing concerns about failing what has been a relatively successful approach in controlling the pandemic to date, i.e., the zero-COVID approach or the zero-tolerance concept that China and a few other countries adopted.

Even though multiple evidence illustrate that the zero-COVID approach can significantly reduce COVID-19 death rates 25 times lower, the negative impacts of the zero-COVID policies are mainly related to health equity issues and socially disadvantaged/marginalized/vulnerable groups. This is particularly seen in low-income countries, making the zero-COVID approach easier to overlook health disparities (1). It is also argued by Jecker and Au (1) that “*when public health focuses on a single disease, overall population health, and core public health suffers, as do core public health values, like equity, solidarity, and justice*” (p. 171). The zero-COVID approach can take up massive public health resources and overpower healthcare systems (2). For instance, to conduct mass testing in the regions under lockdowns, a fair number of medical and healthcare workers are required in a short time suddenly and intensively, which may further lead to additional wastes of time, money, and human resources.

Apart from Shanghai, China's other mega-cities (e.g., Shenzhen, Guangzhou, Beijing, Xi'an, etc.) also encountered a reoccurrence of COVID-19 outbreaks this year. As it appears to date, they have responded more effectively than Shanghai in controlling their situation within a relatively short time. Their control measures included strict lockdown measures and mass testing, which were applied immediately in the early days of their outbreak reoccurrences. This prolonging COVID-19 pandemic has shown that “time” is the most critical factor, providing a promising way to contain and end the pandemic with the least cost and lowest impacts (3). Besides different interpretations and implementations of the national-level zero-COVID policies, emergency responses and the decision-making process of municipal governmental institutions can also be critical factors.

While Shanghai's government refrained from lockdown measures at first, it eventually ended up closing the operations of Asia's largest financial hub. While the early arguments suggested no “one size fits all” policy (4), the situation was worsened due to political pressures (5, 6) and concerns around the economic impacts of Shanghai's lockdown (7). However, it only took a few days for the economic impacts to be seen outside Shanghai's boundaries.

However, Shanghai only imposed a city-wide lockdown from the 3rd of April 2022, after they announced a phased lockdown on the 28th of March 2022 (8). All universities and colleges in Shanghai have been in lockdown since the 12th of March, as 955 new positive cases emerged between March 1st and 15th across the city (9). Later on, over 2,500 cases were recorded on the 27th of March alone (8). On the 22nd of March 2022, Shanghai local authorities announced that they are taking a “slicing and gridding” approach, in which they started screening neighborhoods for infections, as opposed to a city-wide lockdown (10). On the 26th of March, Shanghai authorities emphasized that Shanghai rules out a full city-wide lockdown since “*if the city shuts down, both the whole national economy and global economy will be impacted*.” This was reported at the press conference on epidemic prevention and control (11, 12).

Shanghai's current situation (as of the 4th of April 2022) is associated with indirect consequences, such as “public image,” making the city known as a role model in the prevention and control of COVID-19 (13). Within days of Shanghai's recent lockdown, the national government has stressed strict adherence to the zero-COVID approach to curb the current outbreak at the city level. On the other hand, the international media has taken an immediate standpoint against China's zero-COVID approach and how it is now a failure. The harm caused by such a politicized atmosphere could become more challenging for Shanghai in the combat against the recurring outbreaks that have never been effectively controlled (14). More importantly, the situation is beyond just public health crises (7), challenging all related systems and stakeholders, including governments, health systems, industries, society, and the economy.

Furthermore, there is political pressure to “not reverse course” or “lose face,” which is even more challenging if adaptive measures and reforms could have been allowed. While Shanghai's situation is troubling in many ways, it could lead to subsequent consequences. In this event, if Shanghai fails, then it would appear as the overarching failure of China's zero-COVID approach. In fact, Shanghai may have already failed several battles against the outbreak breaching the city boundaries. It also missed multiple chances to prevent the positive COVID-19 cases from spreading outside to other regions at the first possible best timing. The whole city-wide lockdown was only executed on the 5th of April 2022, mainly after those localized restrictions initiated in March to control the rising positive cases did not work effectively (15). Therefore, if not controlled in a timely manner, the situation could lead to further political pressures concerning how China's approach may change its direction afterward. The current challenges also suggest a hostile take against Shanghai's adaptive measures for the prevention and control of the pandemic, as it is now the foremost image of the zero-COVID approach after the case of Wuhan.

The current situation is complicating further every day as China's zero-policy approach depends on Shanghai's circumstances and experience. If it fails, then the zero-COVID approach may be undermined, and if it succeeds in a short period, it will become a silver lining for potential (and gradual) reforms of the zero-COVID policies. It is now moved to the turning point to reconsider and adjust the zero-COVID policies. The need for a game-changing shift should reflect on the current situations of evolving high variants of this novel coronavirus, increasing social disparities, high rates of public vaccination, declining real economy, etc.

## Author Contributions

AC drafted the paper with the concept. TZ and ZS helped with revision and writing up. All authors contributed to the article and approved the submitted version.

## Funding

AC acknowledges the National Natural Science Foundation of China (NSFC) for the provision of funding for project number 71950410760. He also acknowledges the Ministry of Education, Culture, Sports, Science and Technology (MEXT), Japan Government, and the Network for Education and Research on Peace and Sustainability (NERPS), Hiroshima, Japan.

## Conflict of Interest

The authors declare that the research was conducted in the absence of any commercial or financial relationships that could be construed as a potential conflict of interest.

## Publisher's Note

All claims expressed in this article are solely those of the authors and do not necessarily represent those of their affiliated organizations, or those of the publisher, the editors and the reviewers. Any product that may be evaluated in this article, or claim that may be made by its manufacturer, is not guaranteed or endorsed by the publisher.
